# Participation in rural community groups and links with psychological well-being and resilience: a cross-sectional community-based study

**DOI:** 10.1186/s40359-016-0121-8

**Published:** 2016-04-08

**Authors:** Anthony Lyons, Gillian Fletcher, Jane Farmer, Amanda Kenny, Lisa Bourke, Kylie Carra, Emily Bariola

**Affiliations:** Australian Research Centre in Sex, Health and Society, School of Psychology and Public Health, La Trobe University, 215 Franklin Street, Melbourne, Victoria 3000 Australia; Institute for Human Security and Social Change, La Trobe University, Melbourne, Australia; Rural Health School, La Trobe University, Melbourne, Australia; Rural Health Academic Centre, University of Melbourne, Melbourne, Australia

**Keywords:** Rural, Community participation, Mental health, Well-being, Resilience

## Abstract

**Background:**

Fostering the development of community groups can be an important part of boosting community participation and improving health and well-being outcomes in rural communities. In this article, we examine whether psychological well-being and resilience are linked to participating in particular kinds of rural community groups.

**Methods:**

We conducted a household survey involving 176 participants aged 18 to 94 years from a medium-sized rural Australian town. We gathered data on psychological well-being (Warwick-Edinburgh Mental Well-being Scale), resilience (Brief Resilience Scale), and the types of community groups that people participated in as well as a range of characteristics of those groups, such as size, frequency of group meetings, perceived openness to new members, and whether groups had leaders, defined roles for members, hierarchies, and rules.

**Results:**

Univariable regression analyses revealed significant links between particular group characteristics and individual psychological well-being and resilience, suggesting that the characteristics of the group that an individual participates in are strongly tied to that person’s well-being outcomes. Multivariable analyses revealed two significant independent factors. First, psychological well-being was greatest among those who participated in groups without a hierarchy, that is, equal-status relationships between members. Second, resilience was greater among those who reported having a sense of influence within a group.

**Conclusions:**

Our findings suggest that policymakers wishing to promote participation in rural community groups for health and well-being benefits may do well to encourage the development of particular characteristics within those groups, in particular equal-status relationships and a sense of influence for all group members.

**Electronic supplementary material:**

The online version of this article (doi:10.1186/s40359-016-0121-8) contains supplementary material, which is available to authorized users.

## Background

Approximately 31 % of Australian residents live in regional or rural areas [1]. As in many developed countries, there has been a net population shift in Australia away from rural and remote communities to urban centers, especially in areas that are far from urban centers [2]. Some of this is the result of young people relocating to cities in pursuit of educational and employment opportunities [3]. In some places, it is also due to a restructuring in the agricultural sector resulting in less employment in this sector [2]. One major driver of decline is an historical reduction in the terms of trade in global markets for agricultural products, such as wool and wheat, that have led to lower profits [2]. Industrialization of agriculture has also led to a greater centralization of agricultural production and lower employment [4].

With large numbers of younger people moving to urban centers, many rural areas in Australia have disproportionately older populations. In 2011, approximately 35 % of Australians aged 65 years and older were living in regional and rural areas [5, 6]. Despite this, rural and remote communities tend to have poorer access to health and social support services [7]. Yet, the rapid restructuring of rural Australia has been shown to have a negative impact on the health and well-being of residents living in areas of social and economic decline [2].

In response to these and similar trends globally, policymakers in Australia and internationally have sought innovative and cost-effective strategies to help support the well-being of rural residents, such as initiatives to promote community participation [8] and community co-production of services [9, 10]. Many such initiatives are also aimed at building individual resilience in rural communities to prevent health problems [11]. Although conceptualized in different ways [12], it is generally accepted that resilience, at a minimum, refers to an individual displaying a tendency to bounce back or recover quickly from challenging life events [13]. Resilience can be strengthened through enhanced social relationships [12] and is highly protective of a range of health problems [14].

Psychological well-being and resilience tend to be greater among individuals who report high levels of community involvement and social support [15–18]. Despite major social and economic changes, rural communities are often suggested to have greater community connectedness than urban communities [19], manifested in greater social capital and volunteering [20, 21]. Community groups, such as charities, sports clubs, and arts and hobby groups, bring residents together and provide group members with opportunities to give and receive social support, such as a sense of belonging, practical help, or emotional support [22]. In fact, group involvement has been regarded as a kind of “social cure” in public health, and tends to have its largest impact on psychological well-being and resilience for individuals who participate in groups that they view as important to them or their sense of self and identity [23].

Encouraging the development of thriving community groups offers potential for supporting well-being and preventing mental health problems in rural communities, and is often a priority for policymakers interested in implementing strategies to increase community participation [19]. It is therefore important that policymakers have information on different forms of participation and its links with well-being outcomes. However, little is known about whether particular community group characteristics are associated with higher levels of psychological well-being and resilience than other groups. Whilst there is substantial evidence that well-being outcomes vary when people shift from one group to another [24], it is unclear whether some kinds of groups offer better outcomes than others. It is this knowledge gap that we seek to address in this article.

Community groups can be highly diverse with regard to type, size, structure, and composition. For example, Lickel and colleagues conducted cluster analyses that identified four common group types: task-focused groups; intimacy or social-focused groups; social categories, and; loose associations [25]. Among community groups, task-focused and social-focused groups are the most common. While all groups are inherently social, they vary according to the degree in which they function to fulfil particular tasks or to provide social opportunities for members. Task-focused groups may include charity groups supporting people on low incomes, groups that look after a town’s parks or gardens, or groups that bring people together around a common interest, such as fishing or mountaineering. Social-focused groups may place greater emphasis on the relationships between group members, such as a support group for people who share a common problem or a group that hosts social events.

In addition to group type, groups can vary according to a range of characteristics. Some groups may have many members while others may be small. Some may allow new members to enter the group easily while others may be less open to outsiders. Some may meet often while others less so. In some groups, there may be a particular person who acts as leader. Some may also have prescribed rules of conduct, clearly defined roles for the group members, or hierarchies. Alternatively, there may be groups with a more flexible structure in which people “chip in” when needed rather than having clear roles, hierarchies, or rules. We have been unable to locate any previous studies that have examined whether community groups with differing structures and other characteristics have different levels of well-being among their members.

It could be contended that groups that are highly structured with regard to roles and rules may be better organized at providing social support to their members. Alternatively, groups that are less structured may allow more members to participate in decision-making, potentially providing a greater sense of value and worth. Within some groups, individual members might perceive themselves as having greater levels of influence within a group than other members. Examining perceived influence within a group aligns with research that demonstrates that people are generally happier if they have a sense of control over some or all aspects of their life [26, 27]. Group leaders might naturally perceive themselves as having high levels of influence, but other group members may also feel that they play a central part in shaping the group and its activities. Despite research on the positive benefits of having a sense of control, there is little research on individual’s perceived influence within a community group and links to well-being.

Our study was conducted in a medium-sized rural Australian town in the western region of the state of Victoria, which will remain undisclosed to protect the town’s confidentiality. According to 2011 Australian Bureau of Statistics data, the town consists of 49 % male and 51 % female residents. It has a large older population, with 32 % aged 60 years and older compared to 20 % nationally and a median age of 48 years compared to 37 years nationally. Similar to other rural centers [28], education levels are lower than the national average, with 6 % having a university education compared to 14 % nationally. The town was selected because of relatively high levels of community participation, with approximately 45 % of residents volunteering their time in community groups and initiatives.

We examined the community groups in which residents participated as well as residents’ psychological well-being and resilience. We included a focus on resilience because promoting resilience in rural and other communities has become a major focus for some government and other policymakers. As mentioned earlier, having strong social and other forms of support has also been linked with resilience [17], so it is possible that participating in some community groups enables individuals to draw on support that improves their capacity for resilience. In all, we had three main objectives: 1) to identify the most common groups in which residents participated according to types and characteristics of groups; 2) to identify whether there were links between psychological well-being and participating in groups of particular types and characteristics, and; 3) to identify whether there were links between resilience and participating in groups of particular types and characteristics.

## Methods

### Sample

A total of 176 participants took part in the study. All participants were residents of the town, which consisted of 1298 households and was located approximately 300 km from Melbourne, the capital and largest city in Victoria. Participants in our study were older on average, with an age range of 18–94 years, a mean age of 61.9 years (SD = 14.5), and a median age of 64 years (IQR = 53 to 72 years).

### Survey measures

#### Sociodemographics

Participants indicated their age, gender, employment status (coded as full-time, part-time or casual, not working), country of birth (coded as Australia or overseas), and the number of years in which they had lived in the town. Participants also indicated whether they felt a part of their community (not at all, a little bit, to a moderate degree, very much) and how much influence they believed they had within the community (none, a little, some, a lot; later coded as little or none versus some or a lot).

#### Community group participation

Participants were asked a series of questions about their involvement in community groups. They were specifically asked to think about the group that they felt was most important to them and to answer the questions in reference to this group. This strategy was adopted because residents may be members of multiple groups. The focus on the most important group was based on our reasoning that belonging to this group is likely to have the largest potential effect on psychological well-being and resilience. In other words, the type or characteristics of a group may have less effect on the well-being of individuals for whom the group is only a minor part of their lives or identity, as suggested by previous research on the role of group identity in health and well-being [23].

Participants were asked to indicate the type of group in an open-ended response field, such as noting whether it was a sports group (e.g., tennis club) or support group (e.g., mother’s group). They were then given a list of group types and characteristics and asked to indicate which of the following were true of the group, including whether the group was formally organized, was hierarchical, had strict rules, was relatively relaxed/casual, had a leader, or had each group member’s role clearly defined. They were also able to indicate whether the group was a social or task-focused group. Participants were able to select both options. A variable was therefore computed to indicate whether the group was primarily social-focused, primarily task-focused, or both. Based on questions from a previous study [29], participants were provided with the following statement, “people in this group are very similar to each other”, and agreed or disagreed using a 7-point Likert scale (later coded as either agree or do not agree). Finally, participants indicated how much influence they had within the group (none, a little, some, a lot; later coded as little or none versus some or a lot), the number of group members, the length of time that the group had existed, how often the group gained new members (never, rarely, occasionally, often), and how frequently the group gets together on average (coded as one or more times a week or fortnightly/monthly). See Additional file 1 for the exact wording of questions on community group participation.

#### Psychological well-being

Psychological well-being was assessed with the Short Warwick Edinburgh Mental Well-Being Scale (SWEMWBS) [30], which has strong validity and reliability as a general measure of psychological well-being. It consists of seven items answered on a 5-point scale from “none of the time” to “all of the time”. Examples of items include “I’ve been feeling optimistic about the future”, “I’ve been feeling relaxed”, and “I’ve been dealing with problems well”. Scores are added to produce an overall score between 7 and 35, with higher scores indicating greater psychological well-being.

#### Resilience

Resilience was measured using the Brief Resilience Scale (BRS) [13]. The BRS is a self-reported scale and specifically measures the tendency for an individual to bounce back easily from stressful or challenging life events. It has been linked to a range of health outcomes [14]. The BRS comprises six items answered on a 5-point scale from “strongly disagree” to “strongly agree”. Examples of items include “I tend to bounce back quickly after hard times”, “It does not take me long to recover from a stressful event”, and “I usually come through difficult times with little trouble”. After reversing the scores for negatively worded items, scores are added and averaged to produce an overall mean score between 1 and 5, with higher scores indicating greater resilience.

### Data collection

The survey was conducted between May 2014 and August 2014, and was granted ethical approval from the La Trobe University Human Ethics Committee (Ref: FHEC 13-262). A survey pack containing two hard copy surveys was sent to each of the 1298 households in the town. We chose this method over other alternatives, such as cluster sampling, because the town is relatively small and we wanted to maximize the chance of every resident having access to the survey in order to obtain a sufficiently sized sample. Two copies were sent to each household in case more than one adult lived there, as would be likely for many households. Participation was voluntary and no incentive or reward was offered for participation. Residents who chose to participate first read background information about the study that was attached to the front of the survey. They were informed that only adults aged 18 years or older were permitted to complete the survey and their responses would be anonymous and kept confidential. Those who completed the survey posted it back to the research team using a reply paid envelope. Consent to participate in the study was assumed based on participants voluntarily completing and returning the survey.

### Analysis

Descriptive statistics were used to provide an overview of the sample with regard to demographic variables and the community groups in which participants participated, including each group type/characteristic variable. Associations with psychological well-being were assessed with separate univariable linear regressions conducted for each group type/characteristic variable using scores on the SWEMWBS as the outcome variable. Because mental health often varies according to demographics [31], all regressions controlled for the demographic variables, that is, age, gender, employment status, country of birth, and number of years living in the town (age and years living in the town were included as continuous variables). To identify significant independent factors, variables that were associated with psychological well-being at *p* < 0.15 were entered into a single multivariable regression while controlling for demographic variables. A cut-off of *p* < 0.15 allowed for associations that were not quite significant in the univariable regressions but could be significant in the multivariable regression after taking into account other variables [32]. This same procedure of univariable regressions followed by a multivariable regression was repeated with scores on the Brief Resilience Scale as the outcome variable. In all regressions, standardized (beta) coefficients were computed for each category of a variable and Wald tests assessed the overall effect of each variable. All associations were treated as significant at *p* < 0.05. Stata 11.1 (StataCorp, College Station, Texas, USA) was used to perform all analyses.

## Results

### Sample profile

Table 1 displays numbers and percentages of participants according to each demographic variable. As shown, a majority of participants (63 %) were aged 60 years or older and a little over half (58 %) were female. Close to one half (46 %) of participants were employed in full-time, part-time, or casual work, and 45 % were retired. Almost all were born in Australia (97 %) and three quarters (75 %) had lived in the town for 20 years or more. A large majority (83 %) felt they were moderately or very much a part of their community but more than half (53 %) reported having little or no influence within their community.

**Table 1:** Sample profile (*n* = 176)

	No.	Percent
Gender		
Male	73	42
Female	99	58
Age		
18–39	17	10
40–59	46	27
60+	106	63
Employment status		
Full-time	37	22
Part-time or casual	40	24
Unemployed	12	7
Retired	75	45
Other	4	2
Country of birth		
Australia	169	97
Overseas	5	3
Number of years living in town		
Less than 20 years	43	25
20 years or more	127	75
Feel a part of the community		
Not at all	6	3
A little	23	13
To a moderate degree	61	36
Very much	81	47
Influence within the community		
A little or none	89	53
Some or a lot	80	47

### Community group participation

Of the 176 participants in the study, 160 answered questions on a group that they felt was most important to them. A wide range of groups was reported. Examples included arts and crafts groups, sporting clubs and recreational groups (e.g., cricket club, football club, walking group), volunteer groups (e.g., fundraising groups, emergency services), youth clubs, and senior citizens’ groups. Table 2 displays numbers and percentages of participants reporting on each group type and group characteristic variable in reference to their most important group. Two fifths (39 %) of the groups were primarily social-focused groups. Close to one fifth (19 %) was primarily task-focused. Another one fifth (18 %) was reported as social and task-focused. Close to one quarter (24 %) were neither indicated as social nor task-focused. A majority of participants belonged to groups that were formally organized (81 %) or had a group leader (75 %). Just over one third (36 %) reported belonging to a group that was organized hierarchically. However, most groups were not indicated as having strict rules (63 %) and were relatively relaxed/casual (61 %). Less than half (46 %) had clearly defined roles for the group members.

**Table 2:** Types and characteristics of community groups that participants nominated as their most important (*n* = 160)

	No.	Percent
Group type		
Social	61	39
Task-focused	29	19
Both social and task-focused	28	18
Neither	37	24
Formally organized		
Yes	126	81
No	29	19
Hierarchical		
Yes	56	36
No	99	64
Has strict rules		
Yes	57	37
No	98	63
Relatively relaxed/casual		
Yes	94	61
No	61	39
Has a leader		
Yes	116	75
No	39	25
Group members have clearly defined roles		
Yes	71	46
No	84	54
People in the group are similar to one another		
Agree	90	56
Do not agree	70	44
Participants’ influence within the group		
Little or none	42	27
Some or a lot	114	73
Number of group members		
0–19	56	37
20–49	42	27
50+	55	36
Length of time the group has existed		
Less than 5 years	16	11
Between 5 and 20 years	30	20
More than 20 years	104	69
How often the group gains new members		
Rarely or never	21	14
Occasionally	94	62
Often	37	24
How often group meets		
One or more times a week	83	58
Fortnightly or monthly	58	41
Never meets	2	1

The majority of participants reported that their group had existed for more than 20 years (69 %) and met one or more times a week (58 %). Just over one third (37 %) were in groups of fewer than 20 members while another third (36 %) were in groups of 50 or more members. Three quarters of participants (76 %) indicated that their group gained new members never, or only rarely or occasionally. Only 56 % of participants reported their group having members who were similar to each other. Almost three quarters of participants (73 %) reported having some or a lot of influence within their group. This group was made up of 78 (50 %) participants who reported having some influence within their group and 36 (23 %) having a lot of influence within their group.

### Psychological well-being and community group participation

Table 3 displays mean scores on the SWEMWBS according to the type and characteristics of the groups that participants nominated as their most important. In the univariable analyses, psychological well-being was found to be significantly greater among participants who reported having some or a lot of influence within their group (F[1, 133] = 5.51, *p* = 0.02). This was the only significant factor from the univariable analyses. However, group influence was no longer significant in the multivariable analysis. Instead, group hierarchy was the only significant independent factor. Specifically, psychological well-being was significantly greater among participants in groups that were not organized hierarchically or, in other words, had equal-status relationships (F[1, 120] = 4.33, *p* = 0.04).

**Table 3:** Psychological well-being according to group types and characteristics (*n* = 160)

			Univariable^a^		Multivariable^a^	
	*M*	SD	*β*	*p*	*β*	*p*
Group type				0.06		0.38
Social^b^	26.9	3.2	–		–	
Task-focused	28.4	3.6	0.17		0.08	
Both social and task-focused	27.3	3.0	0.06		0.05	
Neither	25.6	5.1	−0.11		−0.11	
Formally organized				0.19		–
Yes^b^	27.1	3.9	–		–	
No	26.0	3.8	−0.11		–	
Hierarchical				0.07		0.04
Yes^b^	26.0	3.2	–		–	
No	27.5	4.1	0.16		0.19*	
Has strict rules				0.14		0.52
Yes^b^	27.5	3.7	–		–	
No	26.6	3.9	−0.12		−0.06	
Relatively relaxed/casual				0.17		–
Yes^b^	27.3	3.2	–		–	
No	26.3	4.7	−0.12		–	
Has a leader				0.71		–
Yes^b^	27.0	3.8	–		–	
No	26.6	4.2	−0.03		–	
Group members have clearly defined roles				0.12		0.46
Yes^b^	27.3	2.9	–		–	
No	26.6	4.5	−0.13		−0.07	
People in the group are similar to one another				0.65		–
Agree^b^	27.2	3.9	–		–	
Do not agree	26.7	3.9	−0.04		–	
Participants’ influence within the group				0.02		0.08
Little or none^b^	25.6	3.6	–		–	
Some or a lot	27.5	3.9	0.21*		0.16	
Number of group members				0.44		–
0–19^b^	27.0	3.5	–		–	
20–49	27.5	3.2	0.03		–	
50+	26.6	4.7	−0.10		–	
Length of time the group has existed				0.72		–
Less than 5 years^b^	26.2	3.1	–		–	
Between 5 and 20 years	26.9	3.6	0.07		–	
More than 20 years	27.2	4.1	0.11		–	
How often the group gains new members				0.10		0.60
Rarely or never^b^	25.8	3.6	–		–	
Occasionally	26.8	4.1	−0.03		−0.08	
Often	28.0	3.4	0.17		0.02	
How often group meets				0.81		–
One or more times a week^b^	27.2	4.4	–		–	
Fortnightly or monthly	26.8	2.7	−0.02		–	

Results are from univariable linear regressions conducted for each group type and characteristic variable and a single multivariable linear regression involving those variables that were associated with scores on the SWEMWBS at *p* < 0.15 in the univariable regressions. Categories that significantly differ from the reference category are indicated by asterisks. ^a^Adjusted for all demographic variables. ^b^Reference category. *p < 0.05

### Resilience and community group participation

Table 4 displays mean scores on the Brief Resilience Scale according to the type and characteristics of the groups that participants nominated as their most important. In the univariable analyses, resilience was found to be significantly greater among participants who reported their group having strict rules (F[1, 132] = 4.93, *p* = 0.03), and among those who reported having some or a lot of influence within their group (F[1, 134] = 8.27, *p* = 0.005). In the multivariable analysis, only group influence emerged as a significant independent factor (F[1, 122] = 4.39, *p* = 0.04). Being in a group with strict rules was not significantly associated with resilience once other group variables were taken into account.

**Table 4:** Resilience according to group types and characteristics (*n* = 160)

			Univariable^a^		Multivariable^a^	
	*M*	SD	*β*	*p*	*β*	*p*
Group type				0.10		0.45
Social^b^	3.6	0.7	–		–	
Task-focused	4.0	0.6	0.21*		0.11	
Both social and task-focused	3.6	0.7	−0.03		−0.07	
Neither	3.7	0.6	−0.02		0.03	
Formally organized				0.07		0.56
Yes^b^	3.7	0.7	–		–	
No	3.5	0.8	−0.16		−0.06	
Hierarchical				0.38		–
Yes^b^	3.6	0.7	–		–	
No	3.7	0.6	0.08		–	
Has strict rules				0.03		0.30
Yes^b^	3.8	0.7	–		–	
No	3.6	0.7	−0.19*		−0.10	
Relatively relaxed/casual				0.35		–
Yes^b^	3.6	0.7	–		–	
No	3.8	0.6	0.08		–	
Has a leader				0.42		–
Yes^b^	3.7	0.7	–		–	
No	3.7	0.7	0.07		–	
Group members have clearly defined roles				0.17		–
Yes^b^	3.8	0.7	–		–	
No	3.6	0.7	−0.12		–	
People in the group are similar to one another				0.21		–
Agree^b^	3.6	0.7	–		–	
Do not agree	3.8	0.6	0.11		–	
Participants’ influence within the group				0.005		0.04
Little or none^b^	3.4	0.8	–		–	
Some or a lot	3.8	0.6	0.25**		0.19*	
Number of group members				0.42		–
0–19^b^	3.7	0.7	–		–	
20–49	3.8	0.5	0.01		–	
50+	3.5	0.8	−0.11		–	
Length of time the group has existed				0.55		–
Less than 5 years^b^	3.8	0.5	–		–	
Between 5 and 20 years	3.6	0.8	−0.14		–	
More than 20 years	3.7	0.7	−0.09		–	
How often the group gains new members				0.07		0.55
Rarely or never^b^	3.6	0.7	–		–	
Occasionally	3.6	0.7	0.01		−0.02	
Often	3.9	0.6	0.22		0.09	
How often group meets				0.83		–
One or more times a week^b^	3.7	0.7	–		–	
Fortnightly or monthly	3.6	0.7	−0.02		–	

Results are from univariable linear regressions conducted for each group type and characteristic variable and a single multivariable linear regression involving those variables that were associated with scores on the Brief Resilience Scale at *p* < 0.15 in the univariable regressions. Categories that significantly differ from the reference category are indicated by asterisks. ^a^Adjusted for all demographic variables. ^b^Reference category. *p < 0.05 **p < 0.01

## Discussion

Almost all participants in this household survey answered questions on a group to which they belonged and that was most important to them. There was considerable diversity within the groups reported in terms of whether they were social-focused, task-focused or a balance of both, and in terms of group characteristics (e.g., groups with or without leaders, hierarchical structures, strict rules, and clearly defined roles for members). Some groups gained new members often while many were less likely to do so. Some groups were relatively small while others had 50 or more members. Just over half of participants agreed that their group members were similar to one another, while less than half did not agree.

Group type did not emerge as a significant independent factor in the multivariable analyses for either psychological well-being or resilience. It would therefore appear that both social and task-focused groups have similar outcomes in this sample, at least with regard to the measures we included in our study. With regard to group characteristics, only group hierarchy emerged as a significant independent factor, and only for psychological well-being. Participants reported greater psychological well-being if their most important group did not have a hierarchy or, in other words, had relatively equal-status relationships. This was regardless of whether the group was social or task-focused and independent of other group characteristics.

There is little known about the role of equal-status relationships in groups on well-being. Research conducted in whole communities shows poorer health and well-being when there is less equality among community members, and this is attributed in part to stress and lower self-esteem from perceived status differences [33]. It may be that equal-status relationships in community groups produce a perceived sense of fairness where each group member feels included and respected equally. Having little or no hierarchy may also mean that more individuals are able to actively contribute in ways that are personally or socially significant to them. This, however, needs to be tested in further research, perhaps by examining the impact of changes in group structures on subsequent well-being outcomes.

A different pattern emerged for resilience. Group hierarchy appeared not to play a role. Rather, participants who reported having some or a lot of influence within their most important group were significantly more likely to be resilient than those who reported little or no influence. It is probable that having group influence is linked with an individual’s sense of self-efficacy or sense of control over their environment, which is an important component of resilience [26]. It may be that providing opportunities for individuals to have some or a lot of influence within a group that is important to them helps foster resilience. This might also be best achieved in groups that enable equal-status relationships and roles. However, longitudinal research is needed to fully test the relationship between having a great deal of influence within a group and overall resilience. For example, while it is possible that some individuals gain a greater sense of self-efficacy and therefore greater resilience as a result of having influence within their group, it is also possible that some individuals who already display high levels of resilience tend to be elevated to positions in groups that give them influence.

Other research has found that the social groups in which people participate have major implications for their health and wellbeing [23]. Often, people who move out of one group and into another have changes to their health and well-being status, especially if those groups are important to their sense of identity [24]. Our findings provide specific information about the group characteristics most closely linked with well-being and resilience, and especially highlight the importance of having inclusive groups that promote equality and decision-making among all members. Governments, health authorities, and community leaders may wish to consider the potential benefits to community well-being, not only by promoting the formation and maintenance of community groups but particular *kinds* of community group environments. For example, these organizations could consider actively establishing a range of task and social-focused groups for community members to join that each have structures and rules that promote relatively equal-status relationships and enable group members to share decision-making and to have a sense of influence within the group. Groups that enable deeper levels of involvement from members may also be more sustainable, perhaps being more attractive for residents to join and to stay within the group. This is important because achieving sustainable levels of participation is vital to building community resilience [8].

There were several limitations to this study. The study was conducted in a single town, so we do not know whether patterns found in our data are specific to this town or apply across rural centers. Although it would seem unlikely that findings such as the link between well-being outcomes and individuals having influence within their group are not found in other communities, further studies are nevertheless needed elsewhere before drawing firm conclusions.

The sample for the study was skewed toward an older cohort. It is possible that a greater number of older people in the town participate in community groups than younger people because many are retired and may therefore have more time to attend group meetings and to take on group responsibilities. Because many of the questions in the survey targeted experiences of group participation, it is also possible that those who were not participating in community groups were less likely to have finished the survey. That said, our study still included a large proportion of participants younger than 60 years old and our main findings were independent of age. Researchers in the future may wish to consider ways of encouraging younger participants, especially those aged 30 years and younger. Offering incentives may be one possibility. Approaching groups with younger members may be another.

The response rate for the survey was lower than expected. Although the sample was certainly large enough to conduct multivariable analyses, it is possible that those who responded were different to non-responders. One possibility is that responders may have included group leaders. We did not ask participants whether they were leaders within their groups. It is worth noting, however, that 25 % reported belonging to groups that did not have leaders and only 23 % reported having a lot of influence within their group, so it is perhaps unlikely that a large number of participants were group leaders. That said, it is recommended that future studies collect more data on the specific roles that participants have within their groups to see if group role is also a factor in psychological well-being and resilience. Given its moderate sample size, this study should also be considered as a preliminary investigation into links between well-being and group types and characteristics. Further studies ought to be conducted in the future that seek to corroborate our findings using larger samples.

We did not collect data on education or income. It is possible that individuals with greater socioeconomic status are more likely to be awarded roles within groups that give them more influence. We therefore do not know whether there are links between socioeconomic status, group participation, and group influence. We also do not know whether socioeconomic status plays any role in links between well-being and equal-status relationships within groups. It is therefore advisable that future work in this area collects data on socioeconomic status and factors these data into analyses to confirm its role in outcomes.

Group type was classified as primarily social-focused, primarily task-focused, or both. Although we computed a “neither” category for those participants who did not indicate that their group was either social or task-focused, it is not clear what types of groups would fit this category. In some cases, participants may not have fully understood what we meant by social or task-focused, and therefore did not answer at all. However, it is also possible that some participants had a different idea in mind about how to categorize their group. As mentioned earlier, Lickel and colleagues conducted a cluster analysis of groups and identified four broad types [25]. In addition to social-focused and task-focused groups, they identified loose associations (such as people living on the same street) and social categories (such as people having similar ethnic backgrounds). Neither of these types are organized groups as such, and do not appear to apply to the community groups that were the focus of this study. Indeed, social categories may exist without any interaction between people who belong within that category. In any case, only 37 out of 160 participants (who answered questions about group participation) did not indicate that their group was social or task-focused, but it may be worth gathering more information in future research to gain a better understanding of reasons behind particular response patterns to questions such as these and whether some participants have different ideas about how to categorize their group.

Finally, we examined the group that participants nominated as their most important. As mentioned earlier, personally important groups are more likely to have an impact on well-being [23, 24], and this is a strong reason for focusing on participants’ most important group. However, a more complex study of all the groups to which participants belong may provide a more complete picture of the role of different kinds of groups in overall well-being and resilience. For example, based on the findings of the present study, it may be possible that people who belong only to groups with equal-status relationships have better well-being outcomes than those who belong to only one equal-status group and several hierarchical groups. Collecting such data can be challenging if some participants belong to a large number of groups, but this may be worth considering in future research to build a more comprehensive picture of the connections between community group participation and well-being.

## Conclusions

This study examined community group participation and its links with psychological well-being and resilience from a household survey conducted in a medium-sized rural Australian town. Participants focused on their membership of a group that was most important to them. We found that participants with the greatest psychological well-being belonged to groups without hierarchies. We also found that participants with the greatest levels of resilience reported having some or a lot of influence within their group. Our findings also correspond with other research showing close links between community participation, health, and social support. That said, our sample could have been larger. Our sample also consisted mostly of older people. Future studies are therefore recommended that involve larger and more diverse samples before drawing firm conclusions. For now, our findings suggest that specific aspects of the group environment may be linked more strongly with psychological well-being and resilience, and could potentially be considered in policy settings that seek to promote community group participation for improving health outcomes in rural communities.

### Ethics approval and consent to participate

Ethical approval for this study was granted by the La Trobe University Human Ethics Committee (Ref: FHEC 13-262). Consent to participate in the study was assumed based on participants voluntarily completing and returning the survey.

### Consent for publication

Not applicable.

### Availability of data and materials

Data are not available to be shared due to participants consenting to participate in the study on condition that data would not be shared. Additional file 1 has been provided that makes available the exact wording of questions on community group participation.

## Additional file

Additional file 1: Survey questions relating to the participant’s most important group. (PDF 339 kb)

## Abbreviations

BRS: Brief Resilience Scale
SWEMWBS: Short Warwick Edinburgh Mental Well-Being Scale
