# Supplementary material for: Participation in rural community groups and links with psychological well-being and resilience: a cross-sectional community-based study
Source: BMC Psychol. 2016 Apr 8;4:16. doi: 10.1186/s40359-016-0121-8 (PMC4826495; doi:10.1186/s40359-016-0121-8)
Supplement: Additional file 1: — Survey questions relating to the participant’s most important group. (PDF 339 kb) [file 40359_2016_121_MOESM1_ESM.pdf]

Survey Questions Relating to the Participant's Most Important Group

In the space below, please write down the type of group to which you belong that is MOST important to you, that is, the group you had in mind when answering the previous questions. Please do not identify the group. Simply write, for example, "walking group" or "book group". If the group is an organisation, do not give its name. Again, simply indicate the type of group by writing "trade union", "mothers' group", or "tennis club".

Which of the following statements are TRUE of this group: (circle **ALL** that apply)

1 This group is formally organised
2 This group is not formally organised
3 This group is hierarchical (there is a pecking order)
4 This group is non-hierarchical (each group member has equal status in the group)
5 This group has strict rules
6 This group is relatively relaxed / casual
7 This group has a leader(s)
8 Each group member's role in the group is clearly defined
9 This is a social group
10 This is a task-focused group
11 Other _______________
12 None of the above

Again, think about the group that is MOST important to you. Please answer the items below using the scale provided.

	Strongly disagree	Disagree	Disagree somewhat	Neutral	Agree somewhat	Agree	Strongly agree
People in this group are very similar to each other.	1	2	3	4	5	6	7

How much influence do you feel you have within your group?

1 None
2 A little
3 Some
4 A lot

How many members would you say your group has?

1 Less than 10
2 10-19
3 20-49
4 50-99
5 100-199
6 200 or more

For how long has the group existed?

1 Less than 6 months
2 Between 6 months and 1 year
3 Between 1 year and 2 years
4 Between 2 years and 5 years
5 Between 5 years and 10 years
6 Between 10 years and 20 years
7 More than 20 years

How often does the group gain new members?

1 Never
2 Rarely
3 Occasionally
4 Often

Please indicate how frequently the group gets together on average

1 More than once a day
2 Daily
3 Weekly
4 Fortnightly
5 Monthly
6 Yearly
7 Less than yearly
8 The group never meets
